# Immunoreactivity of Gluten-Sensitized Sera Toward Wheat, Rice, Corn, and Amaranth Flour Proteins Treated With Microbial Transglutaminase

**DOI:** 10.3389/fmicb.2019.00470

**Published:** 2019-03-26

**Authors:** Lucilla Scarnato, Gabriele Gadermaier, Umberto Volta, Roberto De Giorgio, Giacomo Caio, Rosalba Lanciotti, Stefano Del Duca

**Affiliations:** ^1^Department of Biological, Geological and Environmental Sciences, University of Bologna, Bologna, Italy; ^2^Department of Biosciences, University of Salzburg, Salzburg, Austria; ^3^Department of Medical and Surgical Sciences, University of Bologna, Bologna, Italy; ^4^Department of Medical Sciences, University of Ferrara, Ferrara, Italy; ^5^Mucosal Immunology and Biology Research Center and Celiac Center, Massachusetts General Hospital Harvard Medical School, Boston, MA, United States; ^6^Interdepartmental Center for Industrial Agro-food Research, University of Bologna, Cesena, Italy; ^7^Department of Agricultural and Food Science, University of Bologna, Cesena, Italy

**Keywords:** transglutaminase, celiac disease, cereal food, gluten-free, sourdough

## Abstract

The aim of this study was to analyze the effects of microbial transglutaminase (mTG) on the immunoreactivity of wheat and gluten-free cereals flours to the sera of patients with celiac disease (CD) and non-celiac gluten sensitivity (NCGS). Both doughs and sourdoughs, the latter prepared by a two-step fermentation with *Lactobacillus sanfranciscensis* and *Candida milleri*, were studied. In order to evaluate the IgG-binding capacity toward the proteins of the studied flours, total protein as well as protein fractions enriched in albumins/globulins, prolamins and glutelins, were analyzed by SDS-PAGE and enzyme-linked immunosorbent assay (ELISA). Results showed that while mTG modified both gluten and gluten-free flour by increasing the amount of cross-linked proteins, it did not affect the serum's immune-recognition. In fact, no significant differences were observed in the immunoreactivity of sera from CD and NCGS patients toward wheat and gluten-free protein extracts after enzyme treatment, nor did this biotechnological treatment affect the immunoreactivity of control samples or the sera of healthy patients. These results suggest that mTG may be used as a tool to create innovative gluten and gluten-free products with improved structural properties, without increasing the immune-reactivity toward proteins present either in doughs or in sourdoughs.

## Introduction

Celiac disease (CD) and non-celiac gluten sensitivity (NCGS) can be regarded as immune-mediated systemic disorders elicited by gluten and related prolamins, among which deamidated gliadins are the most immunogenic proteins (Volta et al., 2013). Epidemiological analysis on populations living in western countries reported that 1% of the European population suffers from CD (Kurien et al., 2016) while the prevalence of NCGS is still far from being established, ranging from 1% (primary care) to 6% (tertiary care) (Volta et al., 2014). Immune-mediated mechanisms are triggered by gluten and evoke intestinal mucosal damage resulting in villous atrophy in patients with CD. This histopathological change is associated with markedly reduced transmucosal absorption ending with nutrient deficiency. Currently, the only therapeutic option for patients with CD is a lifelong strict adherence to a gluten-free (GF) diet (Dowd et al., 2014). Less information is available for NCGS, although some authors have suggested an identical dietary approach for NCGS patients. On the other hand, the quality of baked products, in terms of viscosity, elasticity and cohesion, is strictly dependent on gluten. Indeed, its capacity to create protein aggregates and thus to confer structure and texture to the dough, is a relevant feature for the quality of baked products (Delcour et al., 2012). Producing GF baked goods with preserved palatability and a good nutritional profile is the modern challenge for food technology.

When added to dough, cross-linking enzymes such as transglutaminases (TGs) can create protein networks contributing to the structure of the dough; for this reason, TGs are considered a promising tool for the food industry. TGs are a widely distributed family of enzymes (E.C. 2.3.2.13) (Del Duca and Serafini-Fracassini, 2005), belonging to the class of transferases. Even if TGs from plant and microorganisms lack sequence identity in respect to mammalian ones, they share similar reactions and functions. In fact, either plant or mTGs have conserved cysteine, histidine, and aspartate residues that form the catalytic triad in structurally characterized TGs (Makarova et al., 1999; Serafini-Fracassini et al., 2009).

TGs catalyze three post-translational protein modifications, namely (i) transamidation, (ii) deamidation of endoglutamine residues, and/or (iii) cross-linking between glutamine and lysine residues (intra- or inter-chain), giving rise to protein aggregates (Lorand and Graham, 2003). TGs are also involved in various biological processes and clinical applications (Brunner et al., 2002; Facchiano et al., 2006; Del Duca et al., 2014); further, they are targets in therapeutic developments (Martins et al., 2014; Strop, 2014).

Since the discovery of the first microbial TG (mTG) in 1989 (Ando et al., 1989), many efforts have been made in the selection of mTG-producing strains and in the optimization of the mTG production process (Motoki and Seguro, 1998) with the aim of obtaining low-cost enzymes suitable for food industry applications.

Nowadays, mTG is an important tool in the food industry (Martins et al., 2014) as well as for research and biotechnological applications (Camolezi Gaspar and Pedroso de Góes-Favoni, 2015). Improving firmness, viscosity, elasticity, and water-binding capacity of food products in order to increase organoleptic features, is of great interest in the food industry. Thanks to their protein cross-linking reactions, mTGs have been shown to be a suitable tool for food treatment (Kieliszek and Misiewicz, 2014). Recently, mTG treatment was also shown to improve the structure of GF dough by inducing protein aggregation via protein cross-linking reactions (Scarnato et al., 2016). Moreover, we reported the effects of the combination of mTG and sourdough on the rheological properties, aroma, and shelf-life of wheat bread (Scarnato et al., 2017) and the same food technology has been applied on GF dough, which showed an improved structure (Scarnato et al., 2016). Considering that mTG is not only able to cross-link proteins, but also to catalyze the deamidation of glutamine residue, the latter modification of the mTG-treated products cannot be excluded a priori (Skovbjerg et al., 2004; Gerrard and Sutton, 2005; Heil et al., 2016).

The aim of this research was to evaluate the capacity of mTG to modify wheat and GF proteins by catalyzing protein cross-links and to identify potential changes in the IgG immunoreactivity of those proteins after biothecnological treatment.

## Materials and Methods

### Flours and Doughs Preparation

Barilla S.p.A. (Parma, Italy) provided straight-grade wheat flour; corn, rice and amaranth flours were purchased from local markets. Flour doughs were prepared by mixing flour and water (control dough); sourdough were prepared by adding *L. sanfranciscensis* and *C. milleri* strain to the dough, as described previously (Scarnato et al., 2016; Scarnato, 2017).

### mTG Treatment of Flour Dough Proteins

Ajinomoto kindly provided the mTG, Activa WM (acTG) from to *Streptomyces mobaraensis*, (specific activity: 81–135 U/g, Ajinomoto Foods Europe S.A.S., France).

Treatment of doughs with mTG were carried out by mixing 1U of enzyme/100 mg flour at 40°C for 90 min with constant stirring. Then, treated doughs were stored at −20°C in order to stop mTG-activity or immediately processed for protein extraction.

### Sera Used for Immunological Analysis of Flour Proteins

A collection of sera from blood samples taken for diagnostic purposes was identified in the serum bank of the Clinical Immunology Laboratory (Department of Medical and Surgical Sciences, St. Orsola-Malpighi Hospital of the University of Bologna). The samples included for this study were selected from sera labeled as “CD patients” (*n* = 14), “NCGS patients” (*n* = 17), and “healthy control blood donors” (HCBD) (*n* = 6). IgA anti-TG2 antibodies (TGA), IgG anti-deamidated gliadin peptides antibodies (DGP) and anti-endomysium antibodies (EmA) showed positivity along with the presence of duodenal villous atrophy in patients diagnosed with CD following a gluten-containing diet. NCGS patients were diagnosed following the Salerno Experts Criteria (Catassi et al., 2015). In detail, patients with NCGS are identified as subjects with gluten-related symptoms that rapidly improved after gluten withdrawal and in which CD and wheat allergy have been ruled out. The symptom improvement was considered indicative of NCGS if the score obtained from the modified version of the gastrointestinal symptom rating scale (GSRS), including extra-intestinal symptoms, decreased by at least 30% from baseline after a gluten-free diet (GFD). In addition, positivity for anti-native gliadin antibodies (AGA) of the IgA and/or IgG class, although not specific, is regarded as another tool supporting the NCGS diagnosis. Correct labeling of the selected sera was checked by retesting each sample for TGA, EmA, DGP, AGA, and specific IgE for foods and aeroallergens.

Since we used sera from anonymous blood samples taken for diagnostic purposes, an approval from the St. Orsola-Malpighi Ethics committee was deemed unnecessary. HCBD gave written informed consent prior to blood sampling.

The collection of 36 human sera is listed in Table 1. Pooled sera, containing individual serum from different groups (CD, NCGS, and HCBD), were prepared by adding the same amount of serum from each of the three types.

**Table 1:** Clinical diagnosis and serum antibodies of patients.

**ID**	**Diagnosis**	**EmA IgA**	**tTG IgA**	**DGP-IgG**	**AGA IgA**	**AGA IgG**
1	CD	pos++−	112.8	nd	nd	nd
2	CD	POS++−	31.6	69.1	7.1	29.3
3	CD	POS+++	132.2	144.3	30.8	151.3
4	CD	POS+−−	22.1	68.5	nd	nd
5	CD	nd	139.6	55.6	nd	nd
6	CD	POS+++	116.9	39.6	3.9	42.7
7	CD	nd	135.9	115.9	nd	nd
8	CD	nd	79.4	113.2	nd	nd
9	CD	POS+++	152.8	101.6	28.5	138.9
10	CD	nd	132.8	61.3	nd	nd
11	CD	POS+++	151.9	106.4	19.1	104.2
12	CD	POS+++	150.9	96.4	49.1	139.6
13	CD	POS++−	105.7	nd	nd	nd
14	CD	nd	121.7	17.2	nd	nd
15	NCGS	NEG	4.8	5.1	4.6	40.1
16	NCGS	NEG	4.0	9.5	8.8	114.9
17	NCGS	NEG	5.4	36.0	12.7	65.1
18	NCGS	NEG	5.6	19.7	8.5	34.1
19	NCGS	NEG	4.5	7.9	6.0	47.8
20	NCGS	NEG	5.3	3.4	8.8	44.4
21	NCGS	NEG	5.0	26.0	5.9	85.8
22	NCGS	NEG	5.2	5.3	8.0	41.6
23	NCGS	NEG	5.8	7.6	4.6	51.7
24	NCGS	NEG	4.5	4.5	7.3	50.7
25	NCGS	NEG	4.5	7.0	7.0	87.0
26	NCGS	NEG	5.1	7.6	6.3	40.7
27	NCGS	NEG	2.4	4.1	5.1	54.1
28	NCGS	NEG	5.7	10.0	8.3	43.0
29	NCGS	NEG	5.9	22.8	8.0	119.0
30	NCGS	NEG	4.2	3.3	6.6	54.1
31	NCGS	NEG	5.2	3.4	7.8	88.5
32	HCBD	NEG	1.0	nd	nd	nd
33	HCBD	NEG	0.5	nd	nd	nd
34	HCBD	NEG	1.5	nd	nd	nd
35	HCBD	NEG	1.0	nd	nd	nd
36	HCBD	NEG	0.8	nd	nd	nd

*Coeliac disease (CD, patients 1–14), non-celiac gluten sensitivity (NCGS, patients 15–31), and healthy control blood donors (HCBD, patients 32–36). EMA, anti-endomysial antibodies; tTG, anti-tissue TG antibodies; DGP, anti-deamidated gliadin peptide antibodies; AGA, anti-gliadin antibodies; IgA, immunoglobulin A; IgG, immunoglobulin G; pos+−−, weak positive; pos++−,positive; pos+++, strong positive; neg, negative; nd, not detectable*.

### Serological Tests Performed With Human Sera

Immunoglobulin A tissue transglutaminase antibodies (tTGA) were measured using a commercially available ELISA kit (EuTG IgA, Eurospital, Trieste, Italy), using recombinant human tissue TG as antigen. A cut-off value of 16 arbitrary units (AU), provided by the manufacturer, was adopted.

Immunoglobulin G deamidated gliadin peptide antibodies (DGP) were assessed by ELISA using commercially available kits (a-glia PEP, Eurospital, Trieste, Italy) and an entirely synthetic peptide constructed in a conformational intact manner and then selectively deamidated. According to the manufacturer's instructions, the cut-off value was set at 16 AU.

Immunoglobulin A endomysial antibodies (EmA) were investigated by indirect immunofluorescence using human umbilical cord cryostat sections (4 mm), cut in our laboratory, as substrate. Sera were tested at the initial dilution of 1:5 and, when positive, titrated to the end point.

Immunoglobulin A and Immunoglobulin G anti-gliadin antibodies (AGA) were determined by ELISA using commercially available kits (a-gliatest SIgA and SIgG, Eurospital, Trieste, Italy) and purified a-gliadin as antigen. The cut-off levels, as suggested by the manufacturer, were fixed at 50 and 15 AU for IgG and IgA AGA, respectively.

### Protein Extraction and Dialysis

Proteins from dough, treated with mTG, were extracted with different buffers (1 g of flour/10 mL of buffer) in order to obtain total protein extracts (TE) and fractions enriched in albumins and globulins (F1), prolamins (F2) and glutelins (F3). All steps were carried out at 4°C. To prepare total protein extract, the dough was suspended in 100 mmol/L Tris HCl pH 8.5 containing 20% glycerol and 1.7% β-mercaptoethanol. The mixture was subjected to ultra-sonication for 30 s on ice, and then incubated overnight with constant stirring. The supernatant was collected after centrifugation at 5,000 g for 10 min and then dialyzed against 0.1 mol/L acetic acid for 24 h using 6–8 kDa cut-off dialysis membranes. The protein-enriched fractions were obtained using the same above-described procedure but using different extraction buffers as described by Rallabhandi et al. (2015), with minor modifications. First, the dough was extracted twice with 0.5 mol/L NaCl pH 7.5 for 1 h. The two supernatants, containing the F1-enriched fraction, were pooled before dialyzing against distilled water. The residual extraction pellet was washed with water for 10 min followed by centrifugation. Then, the pellet was resuspended twice in 70% ethanol for 1 h. The extracts containing prolamins (F2) were pooled and the solvent was removed under vacuum. Finally, the glutelin fraction (F3) was extracted by resuspending the residual pellet twice in 50% isopropanol, 1% acetic acid, 0.5% β-mercaptoethanol, followed by dialysis against 0.1 mol/L acetic acid. All the protein extracts were stored at −20°C until further use. The protein content was estimated using Bradford's method with BSA as standards (Bradford, 1976).

### Protein Profile Analysis by SDS-PAGE

SDS-PAGE was performed according to the method of Laemmli (1970). Samples were treated with reducing sample buffer for 5 min at 95°C and then run on 15% SDS-PAGE. Gels were stained with Coomassie brilliant blue R-250 at room temperature and then destained in 10% (v/v) acetic acid. Gels were scanned and analyzed using the Bio-Rad Image Lab 4.0.1 Software.

### Analysis of the IgG-Binding Capacity by ELISA

First, 96-well Maxisorp plates (Nalgene, Rochester, NY) were coated with 50 μL of the protein sample (5 μg/mL) in 150 mmol/L phosphate buffer saline (PBS) overnight at 4°C. The plate was washed twice with PBS and subsequently nonspecific binding sites were blocked with 200 μL/well of 1% BSA in Tris buffer saline containing 0.05% Tween-20 (TBS-T), for 1 h at room temperature. Several serum dilutions (ranging from 1:250 to 1:2,500) were tested in order to identify the optimal number of antibodies for the development of the ELISA analysis. IgG-binding capacity was tested by the addition of 50 μL/well of diluted patient and healthy control sera or pooled sera, respectively, and incubated overnight at 4°C. For each protein sample, blank controls were tested using 0.5% BSA in TBS-T instead of the diluted serum. After four washes, 50 μL/well alkaline phosphatase-conjugated polyclonal anti-human IgG antibody (Thermo Fisher Scientific), diluted 1:1,000 in TBS-T containing 0.5% BSA, was added. After 1 h of incubation at 37°C and another one at 4°C, the plate was washed four times with TBS-T and developed with 50 μL/well of substrate solution, consisting of 10 mmol/L p-nitrophenyl phosphate (PNPP) dissolved in alkaline substrate buffer pH 9.8 (9.7% diethanolamine and 1 mM MgCl_2_). The absorbance was recorded using a microplate ELISA reader at 405 nm (ref. 490 nm). The IgG-binding capacity of each protein sample was characterized by corresponding OD values after background subtraction. The ELISA protocol, coating conditions, reagent dilutions, buffers and incubation times were tested in preliminary experiments.

### Statistical Analysis

All data are reported as means ± SD. Data were analyzed using GraphPad Prism by one-way ANOVA. Differences were considered significant when *p* < 0.05 and very highly significant when *p* < 0.001.

## Results

### Cross-Linking Effect of mTG on Protein Extracts

**Figure 1:**
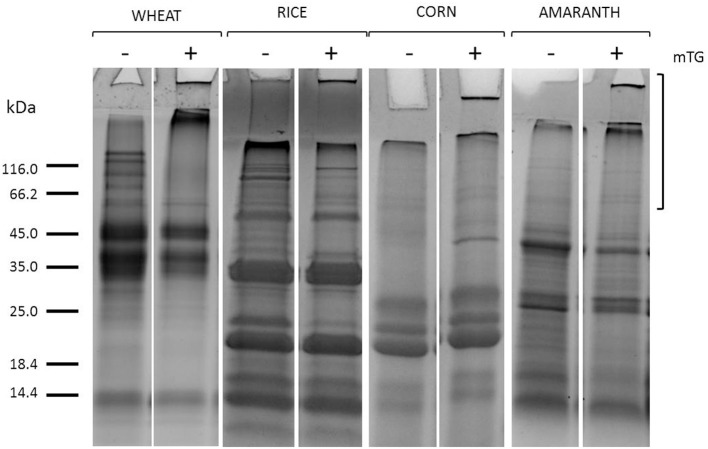
SDS-PAGE of gluten (wheat) and GF flours (rice, corn, amaranth) total protein extracts from control dough before (–) and after (+) mTG treatment. Square brackets show the region in the upper part of the lanes where mTG cross-linked proteins migrate.

The mTG protein cross-linking products were evaluated in control dough, after protein extraction and separation by SDS-PAGE. As shown in Figure 1, after mTG treatment, the protein profiles of gluten-containing dough and GF dough in total protein extracts consisted of the disappearance of some bands along the lanes and the accumulation of protein aggregates. Some protein aggregates were unable to enter the resolving gel, whereas a portion of crosslinked protein does not even enter the stacking gel. These results are the consequence of the cross-links between glutamine and lysine residues of protein substrates catalyzed by mTG (Scarnato et al., 2017).

**Figure 2:**
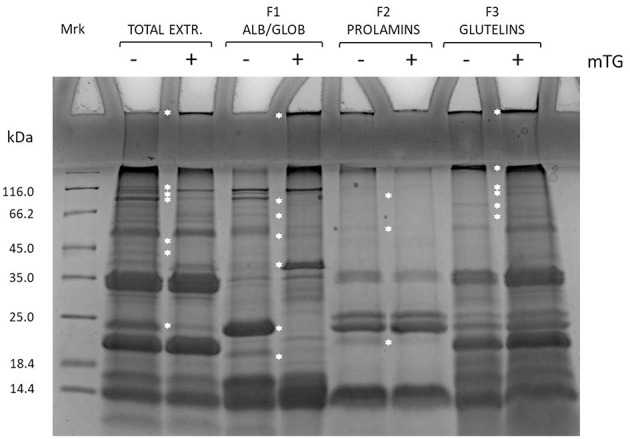
SDS-PAGE of total protein extract and enriched protein fractions (F1, F2, and F3) from rice control dough before (–) and after (+) mTG treatment. Protein-enriched fractions: F1, albumins and globulins; F2, prolamins; F3, glutelins. White asterisk along the lanes highlights bands that change position along the lane after mTG treatment.

The effects of enzyme treatment in the control dough were evaluated also on protein enriched fractions, as shown in Figure 2.

Fractions enriched in F1 and F3 were the main ones involved in protein aggregation because of mTG catalysis of cross-linked products. This has been revealed by the higher accumulation of proteins in the wells and in the running-stacking gel boundary regions in mTG-treated samples as compared with the non-treated ones. Moreover, along the lanes, some bands present in the non-treated sample disappeared in the treated ones, possibly because of protein aggregation to form high molecular weight products. This is particularly evident for the 24 kDa band present in F1 fraction (not treated with mTG) that disappeared when this fraction was treated with mTG. Similar results were also observed either in the other gluten-containing or the GF dough protein extracts (total and enriched fractions) treated with mTG (data not shown). The effect of mTG on sourdough protein extracts from wheat and GF flours has been previously reported (Scarnato et al., 2016, 2017).

### mTG Effects on Flour Proteins Immunoreactivity

**Figure 3:**
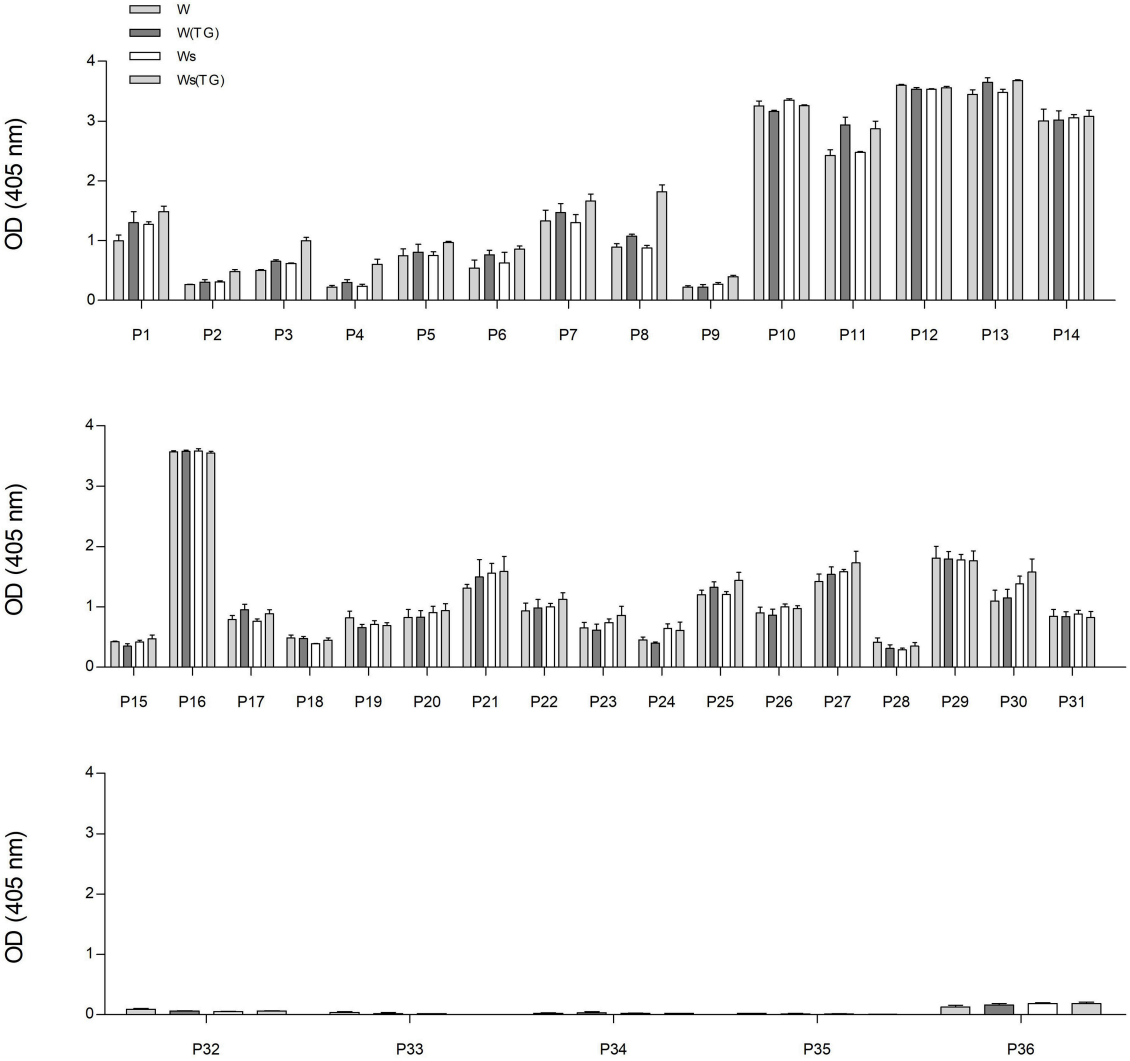
IgG reactivity of sera determined using wheat protein extracts from control dough (W) and sourdough (Ws) treated with mTG (W(TG) and Ws(TG), analyzed by ELISA. From the top: sera from CD patients (from P1 to P14); sera from NCGS patients (from P15 to P31); sera from HCBD (from P32 to P36).

In order to analyze the immunoreactivity of gluten and GF proteins from control dough and sourdough, treated or not with mTG, total protein extracts and F1-, F2-, and F3-enriched fractions were analyzed by checking the IgG-binding capacity of sera from gluten-sensitive patients and healthy blood donors. Single serum IgG reactivity toward total wheat proteins extracted from dough (W) and sourdough (Ws) is shown in Figure 3.

The IgG reactivity distribution of sera reflected the antibody titers of each individual patient. Sera from CD patients and NCGS P16 showed the highest immunoreactivity in terms of OD values, whereas HCBD sera gave no or very low signals.

The graphs in Figure 3 represent the IgG reaction of each individual serum used in the study. There are no significant differences in reactivity within each individual serum toward wheat proteins [all four types: total wheat extract (W), total extract treated with mTG (W(TG)), sourdough (Ws), sourdough + mTG (Ws(TG))].

From the data presented in Figure 3, it is possible to observe that the reactivity of the 36 patients among each other is extremely variable even inside categories of CD, NCGS patients. There is generally a higher IgG reactivity in the CD group, medium reactivity for the NCGS and nearly no reactivity in the healthy controls. This figure is associated with the Table 1, which describes the clinical diagnosis and serum antibodies level of patients.

**Figure 4:**
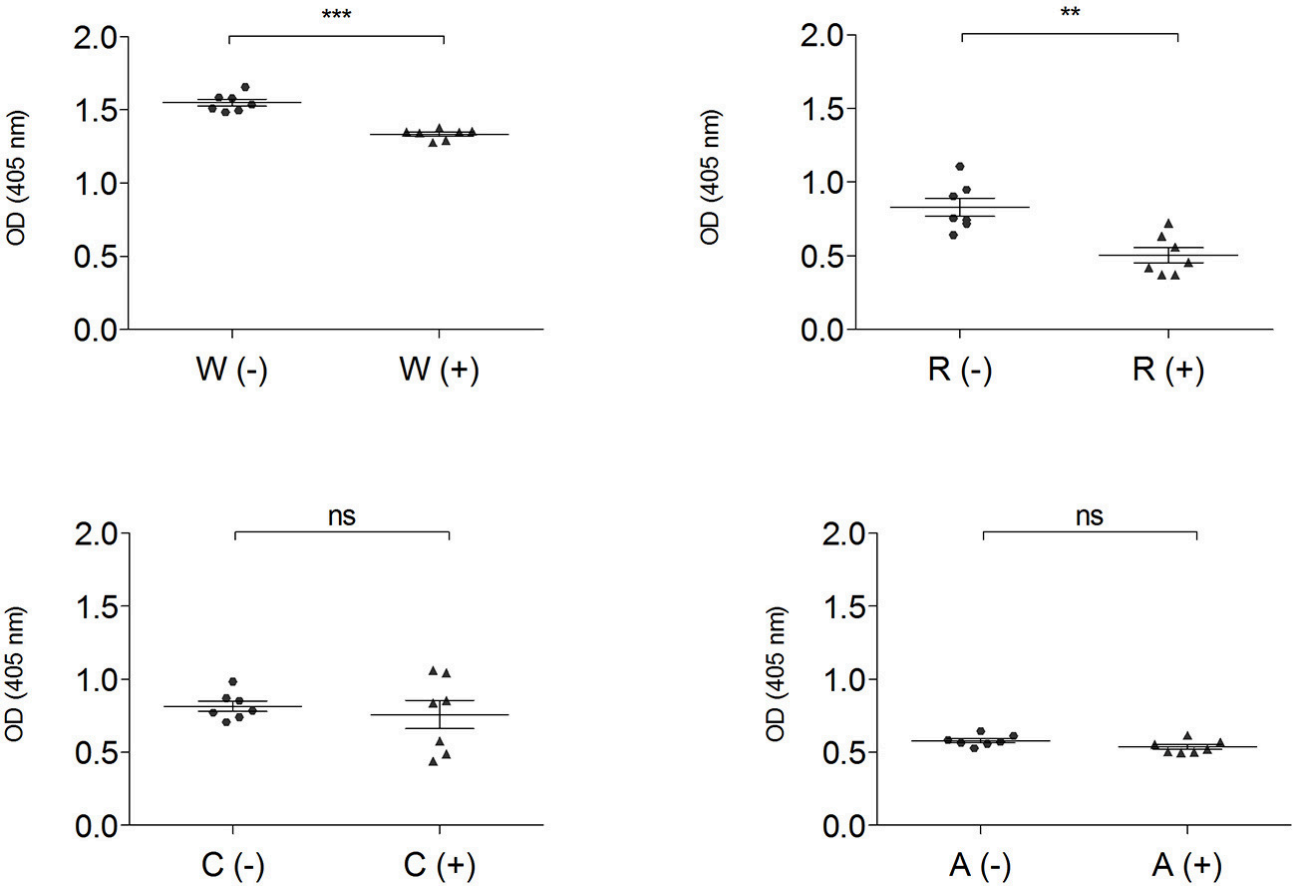
IgG-binding capacity, expressed as OD value of gluten-containing dough and GF flour control dough before (-) ▴ and after (+) • mTG treatment using pooled CD patient' sera (P1–P14). Data are presented as mean values of 7 replicates with significance level: ns = not significant, ^*^p < 0.05, ^**^p < 0.01, and ^***^p < 0.001. W, wheat; R, rice; C, corn; A, amaranth.

The IgG-binding capacity of total protein extracts, before (–) and after (+) mTG treatment, was analyzed using CD patients' pooled sera in order to have a representative trend and to eliminate the variability factor among individuals; data of wheat and GF flours control dough are reported in Figure 4.

Enzymatic treatment did not change the immunoreactivity of total protein extracts both in corn and in amaranth, while in wheat (*p* < 0.001) and rice doughs (*p* < 0.01), mTG treatment significantly decreased the IgG-binding capacity. The OD values represent the level of IgG reactivity to any of the wheat, rice, corn and amaranth proteins present in dough extracts. A higher value indicates that more IgG antibodies against surface-exposed proteins of the flour sources are present in the sera.

**Figure 5:**
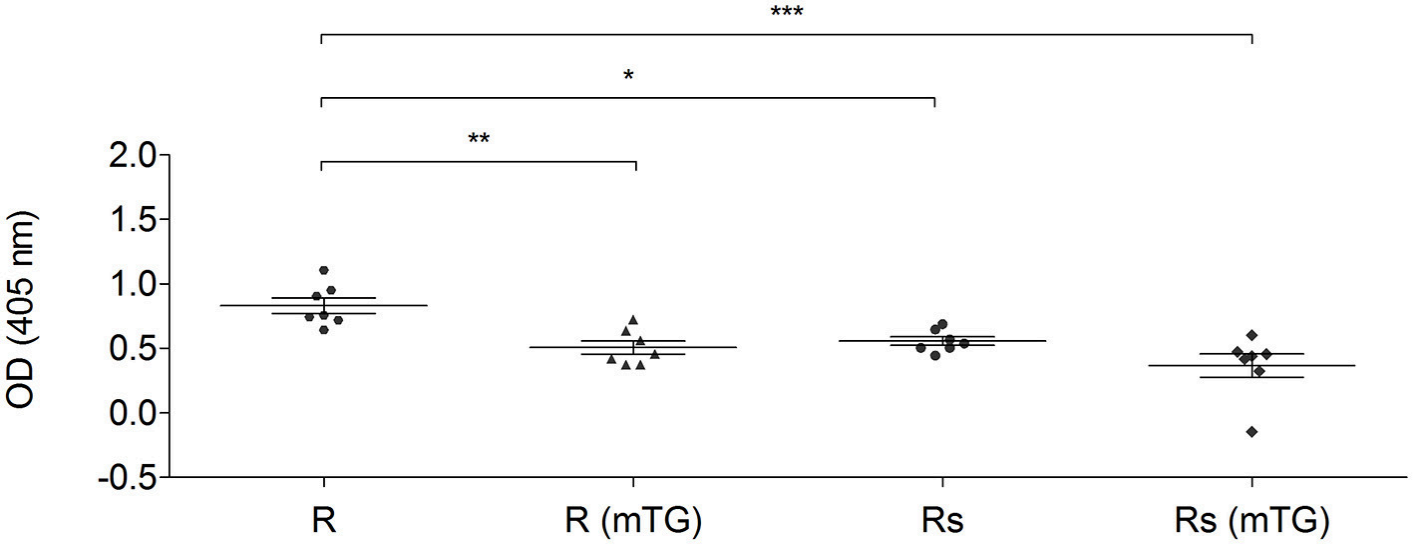
IgG-binding capacity of rice total protein extracts using a pool of sera from CD patients (P1–P14). The protein from rice dough and sourdough were treated with mTG. Data are reported as absorbance at 405 nm of sera diluted 1:250. Data are presented as mean values with significant level: ^*^p < 0.05, ^**^p < 0.01, and ^***^p < 0.001.

The combined effect of mTG and sourdough on GF dough proteins was also analyzed using CD, NCGS, and HCBD pools of sera. Both biotechnological treatments (mTG and sourdough) influenced the immuno-recognition of the sera. As representative data, the total protein extracts from rice doughs, analyzed with the CD patients pool of sera, are shown in Figure 5.

mTG treatment significantly reduced (*p* < 0.01) the specific antibody binding capacity of the protein derived from rice dough. Sourdough fermentation caused a reduction (*p* < 0.05) of the immunoreaction. The statistical analysis did not show any significant differences when the Rs (mTG) sample was compared to the Rs and to R (mTG) samples. In fact, both treatments (sourdough and mTG) resulted in significant antigenicity reduction and the combined treatment did not lead to more decreased antigenicity (Figure 5).

**Figure 6:**
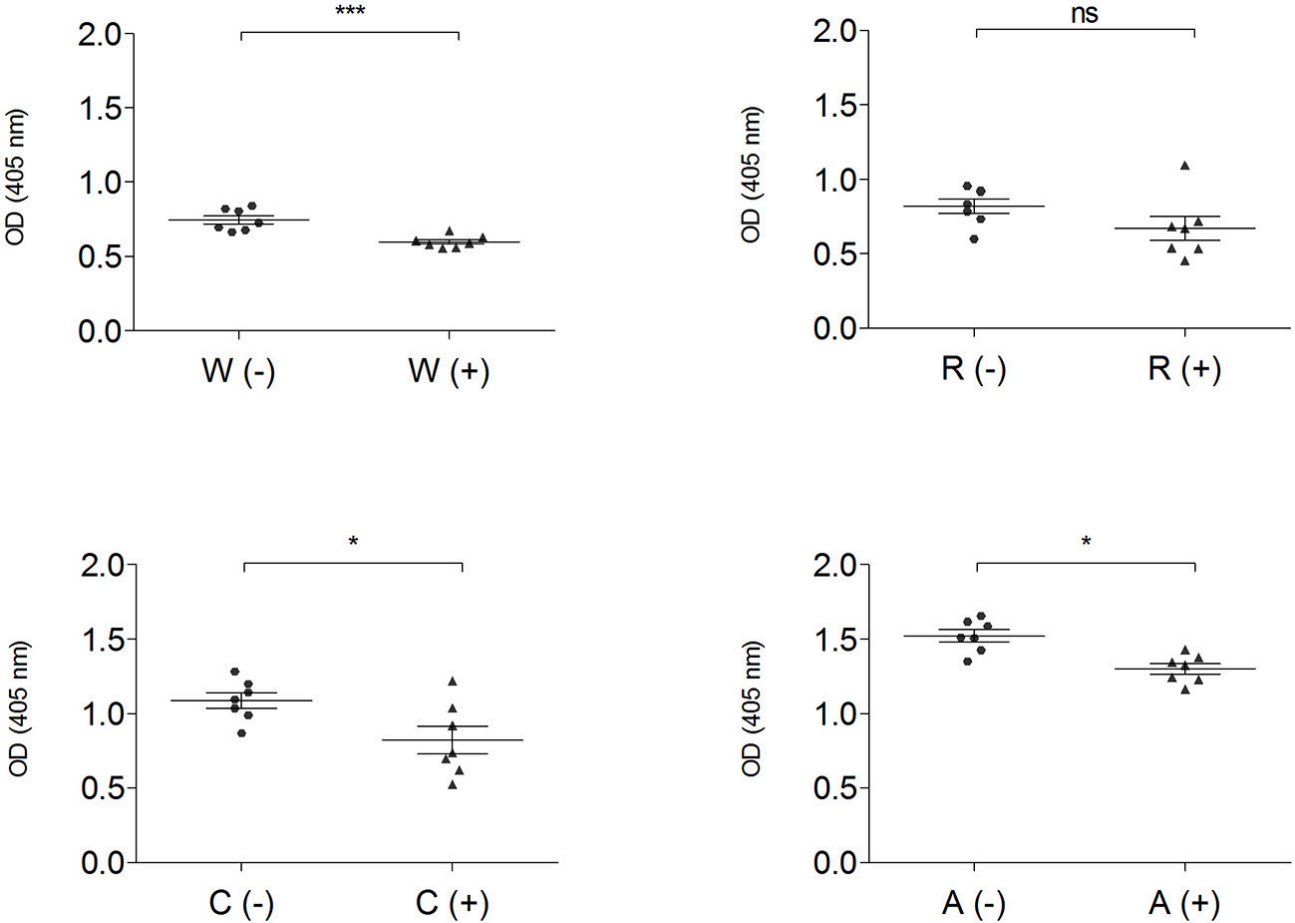
IgG-binding capacity, expressed as OD value of gluten and GF flours before (–) ▴ and after (+) • mTG treatment, determined by ELISA assay using pooled sera from NCGS patients (P15–P31). Data are presented as mean values of 7 replicates with significance level: ns = not significant, ^*^p < 0.05, ^**^p < 0.01, and ^***^p < 0.001. w, wheat; r, rice; c, corn; a, amaranth.

The IgG-binding capacity of total protein extracts from gluten-containing dough and GF dough was measured using pooled sera from NCGS patients (P15–P31) (Figure 6).

This analysis, performed both with wheat and GF doughs, supports the data obtained with CD sera; dough protein immunoreactivity did not increase after mTG treatment. On the contrary, when tested with NCGS sera, mTG treatment decreased immune recognition in doughs of wheat, corn and amaranth (Figure 6). To further evaluate protein IgG reactivity, control dough proteins were extracted by a sequential method using specific buffer solutions. IgG-binding capacity toward F1-, F2-, and F3-enriched fractions, treated or not with mTG, was tested in order to better identify which class of protein showed the highest signal when treated with sera from CD and NCGS patients.

**Figure 7:**
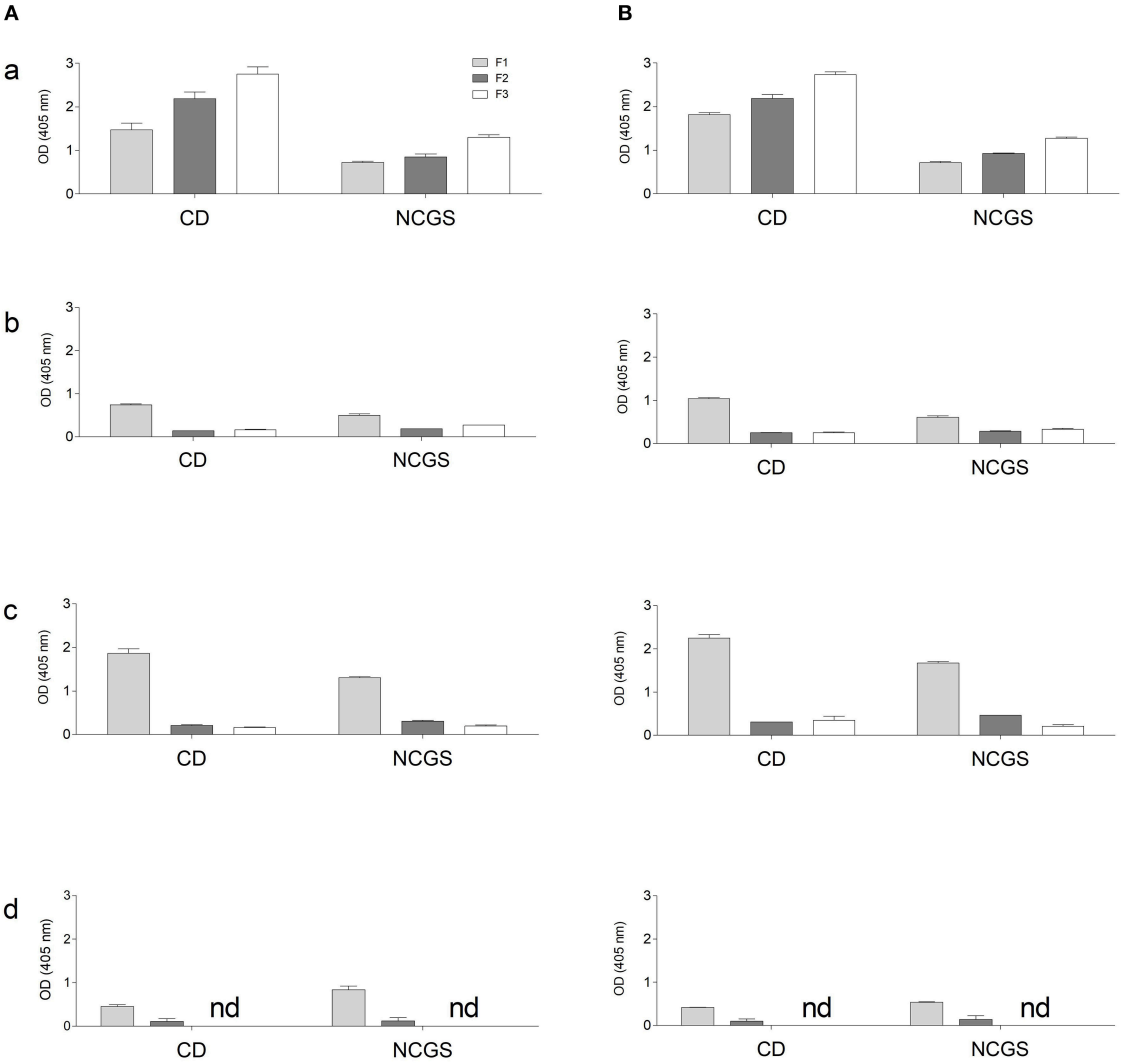
IgG-binding capacity, expressed as OD value of gluten and GF flours dough using pooled sera from CD and NCGS patients, before **(A)** and after **(B)** mTG treatment. Three protein-enriched fractions were analyzed, F1 (albumin and globulins), F2 (prolamins) and F3 (glutelins). Data are presented as mean values ± SD. a, wheat; b, rice; c, corn; d, amaranth.

Results indicate a different immunoreactivity of wheat dough compared to GF dough samples. In fact, wheat protein extracts, and in particular the F3 fraction, showed the highest immunoreactivity, both when tested with CD and NCGS sera. In all the GF samples, immunoreactivity of F2 and F3 fractions was very low and in amaranth F3 did not reach the detection limit of the method. In general, F1 of the GF samples was the only fraction showing considerable immunoreactivity (Figure 7A). In all samples, the CD patients' sera showed a higher immunoreactivity when compared to NCGS patients (Figure 7). Moreover, mTG treatment did not affect the IgG reactivity profile in any of the tested flour doughs.

## Discussion

The lack of structure in bread dough is a difficult challenge while working with GF cereal products. In fact, GF products available on the market are often of low nutritional quality and poor taste. Without gluten, wide ranges of ingredients (i.e., hydrocolloids) are needed to obtain products with organoleptic features appreciated by consumers (Moreira et al., 2013). mTG is proposed as a biotechnological agent to improve the functional properties of structurally poor flour proteins as a processing aid, as it can induce structural protein modifications improving the features of the final product (Camolezi Gaspar and Pedroso de Góes-Favoni, 2015). In our earlier work, GF cereal doughs and sourdoughs made by using *Lactobacillus sanfrancisciensis* and *Candida milleri* were subjected to enzymatic treatment by supplying mTG from *Streptomyces mobaraensis* in order to obtain doughs with an improved texture (Scarnato et al., 2016, 2017). However, the effect of mTG on the immunoreactivity of these doughs was not completely clarified. Therefore, this study was undertaken in order to verify if the mTG reaction affected the immunoreactivity of the treated doughs. In fact, concerns were raised about the use of mTG for flour protein modification as human tissue TG is involved in gliadin deamidation, a key reaction in the etiology of CD. Deamidated gliadins are known to increase immunoreactivity to gluten peptides in CD patients (Sollid, 2000). Moreover, Gerrard and Sutton (2005) suggested that further research was needed to assess this possibility and recommended that TG should not be used in bakery products until this issue is resolved.

Previous studies showed that mTG in combination with sourdough exhibited a positive and synergistic effect by which the excessive hardness and chewiness caused by mTG were counteracted by the sourdough. On the other hand, the protein-aggregating effect of mTG compensated for the proteolytic action of sourdough on protein substrates, which reduces the viscoelastic properties of bread. Data showed that the gluten fraction was the main fraction involved in these cross-links, but in GF flours, mTG was able to exert its action also on the F1 (Albumin and Globulin)-enriched fraction. When mTG activity was checked by microplate assay (while incorporating biotin-cadaverine (BC) on protein substrate immobilized on a microplate), F2 was the main fraction involved in the incorporation of BC, followed by F1-enriched fraction in lentil and amaranth and by F3 (Glutelin)-enriched fraction in rice and corn. The use of sourdough combined with mTG showed a synergistic beneficial effect on bread characteristics, with improved bread rheological features, aroma profile and shelf-life of the baked product. The excessive hardness and chewiness of bread caused by increasing concentrations of mTG were counteracted by the addition of a proper amount of sourdough. On the other hand, the degradative action of sourdough on protein substrates, which reduced the viscoelastic properties of bread, was compensated by the protein-aggregating effect of mTG (Scarnato et al., 2016, 2017).

The aim of the present research was to investigate if the protein modification catalyzed by mTG could affect the immunological features of dough proteins, previously studied for their rheological and organoleptic properties. Data showed that mTG treatment in both gluten and GF flours did not cause a significant increase of IgG-binding capacity. These results provide a perspective in research on GF products, suggesting the possible use of mTG as a biotechnological agent able to create innovative products; its action does not alter the IgG-binding epitopes on substrate proteins.

Under the experimental conditions of our study, mTG protein cross-linking did not affect antibody-binding capacity, thus corroborating results from previous studies. For example, no immunological changes of gliadin extract from pasta dough treated with mTG using the sera of CD patients was observed (Ruh et al., 2014). Interestingly, other data showed that cross-linked gluten flour had a lower immunoreactivity in a rabbit model system, suggesting that the lower deamidation rate of mTG relative to mammalian TGs, together with the cross-linking of gluten peptides, might potentially reduce this risk (Leszczynska et al., 2006). Moreover, transamidation of wheat flour with a food-grade enzyme and an appropriate amine donor can be used to block T cell-mediated gliadin activity and to prevent cereal toxicity (Gianfrani et al., 2007). Immunoblotting using monoclonal antibodies specific to unmodified and/or deamidated gliadin showed no differences between control bread and bread obtained after treatment of the dough with mTG. According to the authors, the concentrations of mTG used in wheat bread preparation do not lead to detectable amounts of deamidated gliadin (Heil et al., 2017).

On the other hand, Berti et al. (2007) demonstrated that mTG-deamidated gliadins increase the IgA antibody reactivity of CD patients with respect to control gliadins (Berti et al., 2007). Others reported an increased immunoreactivity of a CD serum pool to gliadin from bread treated with mTG (Gerrard and Sutton, 2005; Cabrera-Chávez et al., 2008). Recently, Torsten and Aaron (2018) hypothesized that mTG used in food preparation could favor celiac disease initiation and progress. As TGs are also present in plants (Del Duca et al., 2000, 2010; Skovbjerg et al., 2002; Serafini-Fracassini et al., 2009), plant-derived food could be another source for TGs that might reach the reach intestinal tract where these TGs (food derived ones and mTG from microbiota) could play a pathogenic role. This last hypothesis is suggestive but, to our knowledge, not supported by solid experimental evidence.

To settle the question, it has been suggested to test new products by applying the immunoreactivity assay using the sera of CD patients (Cabrera-Chávez et al., 2008). This simple and reliable test could be an easy way to evaluate ingredients and procedures to obtain new GF products and to identify potentially unsafe products. Moreover, further investigations using mouse models could be useful to assess the immunogenicity of mTG doughs and sourdoughs.

In summary, by following the experimental procedure reported in this paper, treatment of gluten and GF flour doughs with mTG leads to an increase in cross-links but does not lead to significant changes in the IgG binding reactivity of the protein extracts with sera from either CD or NCGS patients' sera.

## Author Contributions

LS, GG, and SD conceived the research and experiment design. LS performed the experimental work. UV, RD, and GC collected patient's sera. RL developed and selected the microbial strains for sourdough. All authors contributed in result discussion, manuscript drafting and revision.

### Conflict of Interest Statement

The authors declare that the research was conducted in the absence of any commercial or financial relationships that could be construed as a potential conflict of interest.
